# How can development and plasticity contribute to understanding evolution of the human brain?

**DOI:** 10.3389/fnhum.2015.00208

**Published:** 2015-04-14

**Authors:** Roberto Lent, Fernanda Tovar-Moll

**Affiliations:** ^1^Neuroplasticity Lab, Institute of Biomedical Sciences, Federal University of Rio de JaneiroRio de Janeiro, Brazil; ^2^D'Or Institute of Research and EducationRio de Janeiro, Brazil

**Keywords:** neurons, cortex, evolution, molecular, chemical, development, evo-devo

Humans usually attribute themselves the prerogative of being the pinnacle of evolution. They have large brains with many billion neurons and glial cells (Lent et al., 2012), trillions of synapses and besides all, a plastic hardware that may change either subtly or strongly in response to the external or internal environment (Tovar-Moll et al., 2014). With this hypercomplex apparatus, they are capable of very sophisticated inward computations and outward behaviors that include self-recognition, metacognition, different forms of language expression and reception, prediction of future events, planning and performing long streams of motor acts, subtle emotional feelings, and many other exceedingly complex properties.

The main challenge for research is: how do we explain this gigantic achievement of evolution?

Is it a direct consequence of having acquired a brain larger than our primate ancestors, with huge numbers of computational units? Would it be determined by a particular way these units came to relate to each other, building up logic circuits of powerful capacities? What along development has “made the difference” for the construction of such a complex brain machine? How much of this complexity is innate, how much is sculpted by influence of the external world, by social interaction with our human fellows, and by the history of our own mental trajectory along life?

This special issue of Frontiers addresses some of these intriguing issues. It is comprised of ten reviews by experts in the field.

A reductionist approach is taken by Seth Dobson from Dartmouth College, and Lauren Brent from Duke University, USA. They examine how genomic features of individuals link up to behavioral patterns, in health and disease. Their hypothesis is that polymorphisms of the serotonin transporter gene, typical of primates including humans, offer allelic diversity that make some of us more prone to face adverse social situations (those expressing low levels of the transporter proteins), while others deal better with nonconflictive daily situations (those with high levels). Having two different alleles, therefore, provides long-term benefits to the species to face diverse competition levels within the social group.

Branka Hrvoj-Mihic and her collaborators from the University of California at San Diego, USA, comment about an old suggestion by Greenough et al. (1987) on the two basic mechanisms of plasticity: *experience-expectant plasticity*, by which the brain is provided by development with exuberant hardware (connections, dendrites, synapses), sculpted postnatally to achieve the best configuration for survival; and *experience-dependent plasticity*, associated to the critical periods in development, by which our late-maturing brain allows change and modulation oriented by environmental input. They argue that the brain faces two opposing needs along life: one is to maintain its circuitry functionally stable, the other is to provide it with enough flexibility (=plasticity) to respond appropriately to the environment.

Franco Cauda and his colleagues from the University of Turin, Italy, review the role of an intriguing cortical cell—the von Economo's neuron—described almost 100 years ago (von Economo and Koskinas, 1925). Present in large-brained mammals, including humans, these fusiform neurons are thought to participate in the conscious perception of bodily states, related to the “sentient-self” as proposed by Bud Craig (2010), as well as to differentiation between the self, the others, and the external environment, a strong ability that humans acquired along evolution.

More common and universal than von Economo's neurons are the commissural ones. Commissures are inter-hemispheric connections that exist from lampreys to humans. In the latter, the number of commissures has increased to at least six, and the amount of commissural axons connecting the cerebral hemispheres has reached some hundred millions in humans. This evolutionary trajectory as related to developmental mechanisms is reviewed by Rodrigo Suarez and his colleagues from Queensland Brain Institute, Australia. The corpus callosum, in particular, is the target of their interest, and the knowledge of the developmental events underlying its formation is instrumental to unravel its striking *long-distance plasticity*, as shown in cases of humans born without it (Tovar-Moll et al., 2014).

Using a histological approach, on the other hand, Milos Judas and his colleagues from the Croatian Institute for Brain Research tackled the significance of the cortical subplate as a transient waiting compartment in the developing brain. Situated below the developing cortex, the subplate may possibly be involved in synchronizing and amplifying a period of neurogenesis that gets longer along the evolution of primates, and in relating it with the ingrowing afferent innervation from subcortical regions. Along the same line, Eric Lewitus and his colleagues from the Max Planck Institute of Molecular Cell Biology and Genetics at Dresden, Germany, examine the role of the subventricular zone on cortical folding, characteristic of large brains. They suggest that this region placed adjacent to the earlier ventricular zone becomes more and more complex along evolution, and constrains radial processes and proliferating precursors to assume a conical organization, ending up by mechanically forcing the tissue to fold and generate gyri and sulci.

Leah Krubitzer and James Dooley from the University of California at Davis, USA, take a more systemic approach: they review how the numerous functional areas of the cerebral cortex appear in evolution, related to developmental mechanisms and examples of epigenetic changes on the genome. They comment that cortical expansion follows scaling rules for the different mammalian groups, in line with what was found by Herculano-Houzel et al. (2006, 2007) for the different mammalian orders. The most important issue they tackle here is whether epigenetic influences can be incorporated into the genome and be transmitted across generations. They mention the example of maternal licking and grooming in rats, a behavior that causes increased glucocorticoid receptor transcription persistent along adult life because of a reduction in DNA methylation that can be transferred to the following generation (Kappeler and Meaney, 2010).

Similar to the rat example raised by Krubitzer and Dooley, Louis Lefebvre from McGill University, Montreal, Canada, brings to scene the intriguing examples of social learning that may appear at a given individual, and then prove so useful that becomes rapidly selected by evolution to stay engrained in the species. Even more intriguingly, he reveals that the same phenomenon was observed in tits (Fisher and Hinde, 1949) and chimpanzees (Kawai, 1965): convergent evolution of high cognitive abilities?

Ricardo Garcia and his collaborators from Universidad de Chile, Universidad del Desarrollo and Pontificia Universidad Catolica de Chile, tackle an even more complex cognitive ability, supposedly characteristic of humans: language. They review in detail the intricate circuits of monkey and human brains, point out similar features between them, and propose a “trajectory” for the evolution of language, from imitation of hand movements with communicative meaning, to a more complex system of manual and facial pantomimes, and finally a protospeech that opened way to full language.

Finally, Michael Anderson and Barbara Finlay, from the University of Maryland and Cornell University, USA, wrap up data on brain development, plasticity and evolution, providing a deep, broad, historical review about the concept of modularity of brain organization. They also end up by questioning if brain evolution has really been made possible only by increase or decrease of modules (neurons, connections, functional regions etc.), or if, alternatively, existing basic modules are simply reused in different ways to provide diversity in animal behavior and cognitive abilities.

## Conflict of interest statement

The authors declare that the research was conducted in the absence of any commercial or financial relationships that could be construed as a potential conflict of interest.
